# Trajectories of (Bio)markers During the Development of Cognitive Frailty in the Doetinchem Cohort Study

**DOI:** 10.3389/fneur.2019.00497

**Published:** 2019-05-31

**Authors:** M. Liset Rietman, Gerben Hulsegge, Astrid C. J. Nooyens, Martijn E. T. Dollé, H. Susan J. Picavet, Stephan J. L. Bakker, Ron T. Gansevoort, Annemieke M. W. Spijkerman, W. M. Monique Verschuren

**Affiliations:** ^1^National Institute for Public Health and the Environment, Bilthoven, Netherlands; ^2^Julius Center for Health Sciences and Primary Care, University Medical Center Utrecht, Utrecht University, Utrecht, Netherlands; ^3^Department of Public and Occupational Health, Amsterdam Public Health Research Institute, Amsterdam UMC, Vrije Universiteit Amsterdam, Amsterdam, Netherlands; ^4^Department of Molecular Medicine, University of Texas Health Science Center at San Antonio, San Antonio, TX, United States; ^5^Department of Internal Medicine, University Medical Center Groningen and University of Groningen, Groningen, Netherlands

**Keywords:** cognitive frailty, markers, biomarkers, trajectories, Doetinchem Cohort Study

## Abstract

**Background:** Long-term changes in (bio)markers for cognitive frailty are not well characterized. Therefore, our aim is to explore (bio)marker trajectories in adults who became cognitively frail compared to age- and sex-matched controls who did not become cognitively frail over a 15 year follow-up. We hypothesize that those who become cognitively frail have more unfavorable trajectories of (bio)markers compared to controls.

**Methods:** The Doetinchem Cohort Study is a longitudinal population-based study that started in 1987–1991 in men and women aged 20–59 years, with follow-up examinations every 5 years. For the current analyses, we used data of 17 potentially relevant (bio)markers (e.g., body mass index (BMI), urea) from rounds 2 to 5 (1993–2012). A global cognitive functioning score (based on memory, speed, and flexibility) was calculated for each round and transformed into education and examination round-adjusted z-scores. The z-score that corresponded to the 10th percentile in round 5 (z-score = −0.77) was applied as cut-off point for incident cognitive frailty in rounds 2–5. In total, 455 incident cognitively frail cases were identified retrospectively and were compared with 910 age- and sex-matched controls. Trajectories up to 15 years before and 10 years after incident cognitive frailty were analyzed using generalized estimating equations with stratification for sex and adjustment for age and, if appropriate, medication use. Results were further adjusted for level of education, depressive symptoms, BMI, and lifestyle factors.

**Results:** In men, (bio)marker trajectories did not differ as they ran parallel and the difference in levels was not statistically significant between those who became cognitively frail compared to controls. In women, total cholesterol trajectories first increased and thereafter decreased in cognitively frail women and steadily increased in controls, gamma-glutamyltransferase trajectories were more or less stable in cognitively frail women and increased in controls, and urea trajectories increased in cognitively frail women and remained more or less stable in controls. Results were similar after additional adjustment for potential confounders.

**Conclusions:** Out of the 17 (bio)markers included in this explorative study, differential trajectories for three biomarkers were observed in women. We do not yet consider any of the studied (bio)markers as promising biomarkers for cognitive frailty.

## Introduction

Frailty is a state of increased vulnerability to adverse health outcomes when exposed to stressors caused by the cumulative decline in one or more domains of functioning, including the cognitive domain (1–4). Moderate cognitive decline is part of the normal aging process (5). Some elderly are confronted with accelerated cognitive decline, which could eventually lead to (mild) cognitive impairment or dementia. A (reversible) state of cognitive vulnerability within mild cognitive impairment has been termed “Cognitive frailty” (6). Although the existence of cognitive frailty and its definition are still under debate (7), there seems to be broad agreement that cognitively frail people experience accelerated cognitive decline (i.e., cognitive dysfunction) without having a form of dementia (8). In this study, people were considered to be cognitively frail when their global cognitive functioning was poor, given their level of education.

It is not yet fully understood how cognitive frailty develops and whether it can be detected at an early stage. However, there are indications that processes of inflammation and oxidative stress are involved and that C-reactive protein (CRP) could potentially serve as a biomarker (9). In addition, in previous studies we observed associations between body mass index (BMI), self-reported health, several biomarkers (e.g., β-cryptoxanthin and zeaxanthin), and cognitive frailty (10, 11). Unfavorable changes in these and other (bio)marker levels may precede cognitive frailty. Studying these changes can provide insight into the molecular pathways involved and could point out promising biomarkers for cognitive frailty.

In the Doetinchem Cohort Study (DCS), various markers (e.g., self-reported health, BMI) and biomarkers (e.g., CRP, urea), have been measured over a time span of at least 15 years (12). Out of these (bio)markers, we identified 17 possibly relevant (bio)markers for cognitive frailty. These are mainly cardiometabolic, inflammatory, and oxidative stress markers. These types of markers have been linked to cognitive decline (13) and could therefore possibly serve as biomarkers for cognitive frailty. Since cognitive frailty arises gradually, it is meaningful to study how (bio)markers “behave” in the course of developing cognitive frailty. These insights can be helpful for the development of treatment and prevention. Therefore, our aim is to explore the trajectories of several (bio)markers during the development of cognitive frailty in adults and compare these to the trajectories of age- and sex-matched controls. We hypothesize that those who become cognitively frail have more unfavorable trajectories of (bio)markers compared to controls.

## Methods

### Cohort

The DCS is a longitudinal population-based cohort study starting in 1987–1991 (round 1) examining 7,769 men and women aged 20–59 years living in Doetinchem, a town in the Netherlands. Adults who participated in the first round were invited for follow-up examinations in 1993–1997 (round 2, *n* = 6,117, mean age: 46 years), 1998–2002 (round 3, *n* = 4,918, mean age: 51 years), 2003–2007 (round 4, *n* = 4,520, mean age: 56 years), and 2008–2012 (round 5, *n* = 4,018, mean age: 60 years). Response rates were 75% or higher in all rounds. Verschuren et al. (14) and Picavet et al. (12) have described the study design in more detail. All participants gave written informed consent in each round and the study was approved by the Medical Ethics Committee of the University Medical Center Utrecht.

### Incident Cognitive Frailty

Cognitive functioning was assessed among participants aged ≥45 years. Trained personnel carried out the cognitive tests according to a standardized protocol. In rounds 2–5, global cognitive functioning was measured with a neuropsychological test battery assessing three domains: memory function, information processing speed and cognitive flexibility. These were tested using the 15 Words Verbal Learning Test (VLT) (immediate and delayed recall) (15), the Stroop Color–Word Test (16), the Word Fluency Test (17), and the Letter Digit Substitution Test (18). Nooyens et al. (19) have described the cognitive tests in more detail. From the separate test scores, one global cognitive functioning score was calculated for each round. Next, the global cognitive functioning scores were transformed into z-scores, based on the mean and standard deviation in round 5, and were adjusted for level of education and for the number of tests performed during follow-up to take a possible learning effect into account. The z-score that corresponded to the 10th percentile in round 5 (z-score = −0.77) was applied as cut-off point for incident cognitive frailty in rounds 2, 3, 4, and 5. This is consistent with the definition used in one of our previous studies, where we also defined people as being cognitively frail when their global cognitive functioning was poor, given their level of education (11). Since the prevalence of frailty naturally increases with age, this was not included in the definition of cognitive frailty. Participants with a score below the cut-off point were considered incident cognitively frail and participants with a score above this value were considered not cognitively frail. As cognitive tests were only performed among participants aged ≥45 years, we defined participants < 45 years as not being cognitively frail. Participants aged ≥45 years without data on cognitive functioning were excluded.

### Measurements

#### Markers

Weight and height (to calculate the BMI), waist circumference, and diastolic and systolic blood pressure were measured according to standard protocols (14). Standardized questionnaires were used to obtain data on self-reported health, depressive symptoms (assessed with the Mental Health Inventory-5 and the Vitality dimension of the 36-Item Short-Form Health Survey) (20, 21), level of education, smoking status, alcohol consumption, physical activity (categorized using the Cambridge Physical Activity Index) (22), use of anti-hypertensive medication, cholesterol-lowering medication, and glucose-lowering medication.

#### Biomarkers

Total and high-density lipoprotein (HDL) cholesterol were measured with standardized enzymatic methods. In 2013–2014, standardized enzymatic methods were used to retrospectively determine triglycerides, alanine aminotransferase (ALT), gamma-glutamyltransferase (GGT), high sensitivity CRP, albumin, uric acid, cystatin C, and creatinine of rounds 2–5 using blood plasma that had been stored in freezers. Participants with only one measurement of the (bio)markers were excluded and participants who were pregnant in a particular round were excluded for that round only. Details of all measurements are described in the Appendix A (Supplementary Material).

### Statistical Analyses

#### Matching

The time of incident cognitive frailty was the first examination round in which participants scored below the cut-off point of −0.77. From this point in time, we were able to investigate trajectories with a maximum of 15 years before and 10 years after incident cognitive frailty. There were not enough observations to study the trajectories up to 15 years after incident cognitive frailty. Both cognitive frailty and biomarker levels vary strongly by sex and age. Therefore, we used a matching design in which each incident cognitively frail person was matched to two controls based on sex, age (with 5-year age categories), and examination round. We excluded two incident cognitively frail cases, as no suitable controls could be identified.

#### (Bio)marker Trajectories

Trajectories of (bio)markers for incident cognitively frail cases and controls were analyzed using generalized estimating equations (GEE) with an unstructured correlation structure. The GEE analysis was performed for each (bio)marker (dependent variable) separately and with cognitive frailty (“yes” vs. “no”) as the main determinant. This resulted in separate estimates (i.e., adjusted marginal means) at each point in time for cases and controls. With these estimates, trajectories for the cognitively frail and the controls were constructed. This approach is consistent with the method used by Hulsegge et al. (23).

Analyses were stratified for sex and the model included age (linear and quadratic), examination round (categorical variable with round 5 as reference category), and time (categorical variable). Time consisted of six categories ranging from T_−15_ to T_+10_ with T_0_ as the moment of incident cognitive frailty. Age was centered at 60 years because this was approximately the mean age at round 5, which resulted in (bio)marker levels of someone who was hypothetically 60 years old. Trajectories of systolic and diastolic blood pressure were adjusted for self-reported anti-hypertensive medication, trajectories of total cholesterol, HDL cholesterol and triglycerides were adjusted for self-reported cholesterol-lowering medication, and trajectories of glucose were adjusted for the self-reported use of glucose-lowering medication.

Triglycerides, ALT, GGT, and CRP had a skewed distribution. Therefore, we log-transformed these biomarkers and reported back-transformed geometric means. Differences between trajectories of cognitively frail people and their controls were tested using an overall interaction term between cognitive frailty and time, and a *p*-value lower than 0.1 was considered statistically significant. This was obtained via the joint tests for GEE, where differences in slopes were calculated based on five interaction terms (frailty^*^T_−15_, frailty^*^T_−10_, frailty^*^T_−5_, frailty^*^T_+5_, frailty^*^T_+10_) with T_0_ (i.e., moment of incident cognitive frailty) as reference category.

To summarize, trajectories up to 15 years before and 10 years after incident cognitive frailty were analyzed and compared to controls using GEE with stratification for sex and adjustment for age and, if appropriate, medication use (model 1). We verified whether the results changed after additional adjustment for level of education, depressive symptoms (model 2), BMI, smoking, alcohol intake, and physical activity (model 3). Trajectories of BMI and waist circumference were not adjusted for BMI.

We performed a sensitivity analysis to study the potential impact of loss to follow-up due to mortality on our results. To this end, we excluded participants who died during the follow-up period (1993–2012) along with their matched case and/or control(s) and compared the results to those obtained in the total population. All analyses were performed using SAS 9.4 software (SAS Institute, Cary, North Caroline, USA).

## Results

### Population Characteristics

After excluding participants ≥45 years without data on cognitive functioning and applying the additional exclusion criteria, 5,139 participants remained for further analyses. Over the course of the study, 6 participants were defined as incident cognitively frail in round 2, 116 participants in round 3, 134 participants in round 4, and 202 participants in round 5. In total, 455 participants became cognitively frail of which 303 (67%) were men and 152 (33%) were women. At incident cognitive frailty (T_0_), men had an average age of 65.5 (SD 7.6) and women of 66.9 (SD 7.8). Cognitively frail people more often had a low level of education and a slightly higher BMI, waist circumference, and systolic blood pressure than controls (Table 1). In addition, cognitively frail men and women more often reported to have poor or fair health and their medication use was higher compared to controls.

**Table 1:** Population characteristics for incident cognitively frail men and women and their controls at T_0_.

	**Men**		**Women**	
	**Controls *N* = 606**	**Cognitively frail *N* = 303**	**Controls *N* = 304**	**Cognitively frail *N* = 152**
**SOCIO-DEMOGRAPHIC FACTORS**				
Age (years), mean (SD)	63.0 (7.8)	65.5 (7.6)	64.6 (8.0)	66.9 (7.8)
Low level of education, %	42	49	62	70
**LIFE-STYLE FACTORS**				
Current smoker, %	15	24	17	14
Alcohol consumption (1 or more glasses/week), %	77	76	60	39
**MEDICATION USE**				
Anti-hypertensive, %	24	27	29	34
Cholesterol-lowering, %	17	22	16	20
Glucose-lowering, %	6	10	6	7
**HEALTH**				
Poor or fair self-reported health, %	16	23	22	34
Mental health (range 0–100), mean (SD)	81.3 (14.3)	78.2 (15.9)	75.6 (15.4)	70.2 (17.3)
Vitality (range 0–100), mean (SD)	71.0 (17.4)	68.9 (18.0)	65.8 (17.4)	59.9 (19.8)
**ANTHROPOMETRIC DATA**				
BMI (kg/m^2^), mean (SD)	26.9 (3.5)	27.4 (3.7)	27.1 (4.7)	28.3 (5.5)
Waist circumference (cm), mean (SD)	100.7 (10.0)	102.7 (10.5)	93.5 (12.0)	97.4 (13.3)
Systolic blood pressure (mmHg), mean (SD)	134.7 (17.2)	138.0 (18.6)	133.8 (18.5)	137.8 (19.4)
Diastolic blood pressure (mmHg), mean (SD)	81.9 (9.8)	81.4 (9.9)	79.9 (9.7)	80.4 (10.1)
**BIOMARKERS**				
Total cholesterol (mmol/L), median (IQR)	5.5 (4.8–6.1)	5.3 (4.4–6.3)	5.9 (5.1–6.7)	5.9 (5.4–6.5)
HDL cholesterol (mmol/L), median (IQR)	1.21 (1.00–1.44)	1.17 (0.98–1.42)	1.51 (1.23–1.81)	1.39 (1.17–1.67)
Glucose (mmol/L), median (IQR)	5.3 (4.8–6.1)	5.4 (4.9–6.1)	5.2 (4.8–5.8)	5.3 (4.9–5.9)
Triglycerides (mmol/L), median (IQR)	1.45 (1.06–2.07)	1.55 (1.10–2.08)	1.40 (1.05–1.90)	1.54 (1.11–2.10)
ALT (U/L), median (IQR)	18 (14–24)	18 (14–24)	16 (12–20)	14 (11–18)
GGT (U/L), median (IQR)	28 (19–40)	27 (20–41)	19 (15–29)	19 (14–26)
CRP (mg/L), median (IQR)	1.17 (0.62–2.40)	1.61 (0.81–3.07)	1.38 (0.68–2.89)	1.50 (0.70–2.48)
Albumin (g/L), median (IQR)	45 (43–46)	45 (43–46)	45 (43–47)	45 (43–46)
Cystatin C (mg/L), median (IQR)	0.86 (0.78–0.97)	0.91 (0.81–1.02)	0.81 (0.72–0.94)	0.89 (0.79–0.99)
Creatinine (umol/L), median (IQR)	84 (76–93)	83 (76–91)	67 (60–74)	69 (62–77)
Uric acid (mmol/L), median (IQR)	0.34 (0.29–0.38)	0.33 (0.29–0.38)	0.27 (0.23–0.30)	0.27 (0.22–0.32)
Urea (mmol/L), median (IQR)	6.2 (5.4–7.1)	6.2 (5.3–7.4)	5.8 (4.9–6.6)	6.1 (5.1–7.2)

*BMI, body mass index; HDL cholesterol, high-density lipoprotein cholesterol; ALT, alanine aminotransferase; GGT, gamma glutamyltransferase; CRP, C-reactive protein*.

### (Bio)marker Trajectories

**Figure 1:**
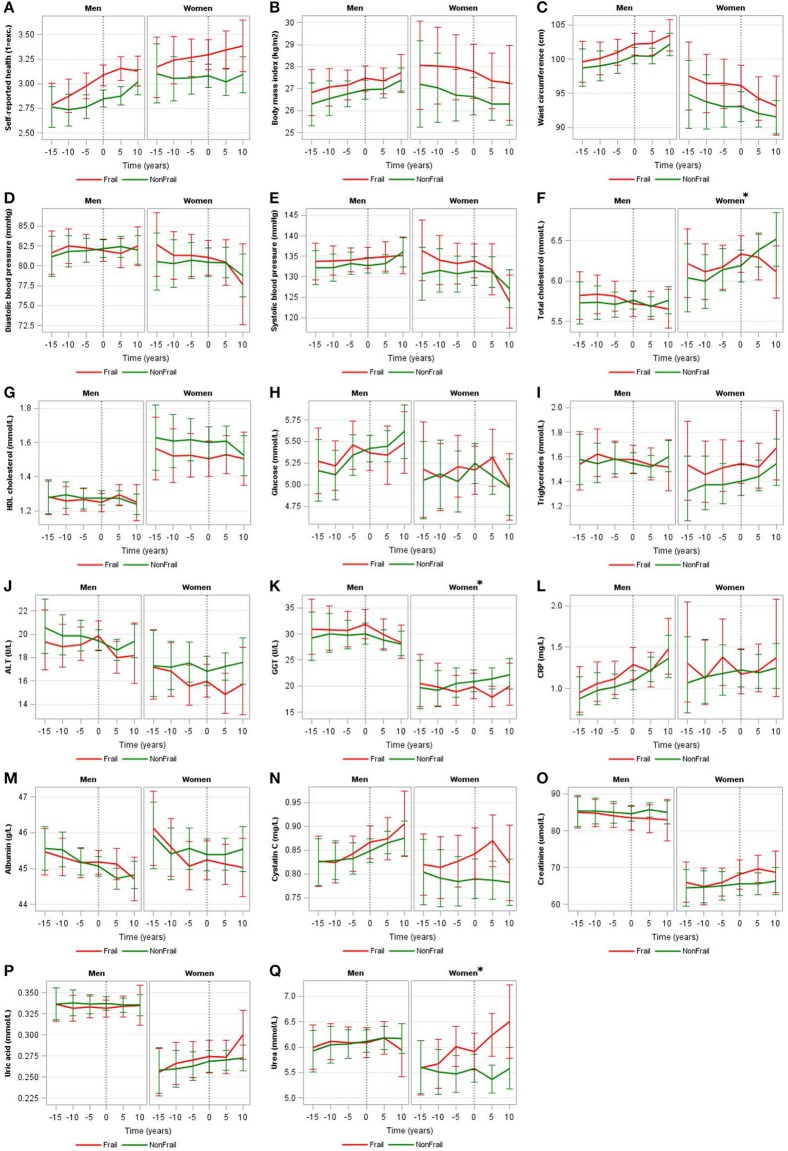
Trajectories of (bio)markers for incident cognitively frail men and women and their controls. Trajectories of self-reported health **(A)**, body mass index **(B)**, waist circumference **(C)**, diastolic blood pressure **(D)**, systolic blood pressure **(E)**, total cholesterol **(F)**, HDL cholesterol **(G)**, glucose **(H)**, triglycerides **(I)**, ALT **(J)**, GGT **(K)**, CRP **(L)**, albumin **(M)**, cystatin C **(N)**, creatinine **(O)**, uric acid **(P)**, and urea **(Q)** of incident cognitively frail people (red lines) and controls (green lines) with 95% confidence intervals stratified by sex and corrected for age and, if appropriate, medication use (model 1), where men and women were hypothetically 60 years old at the time of incident cognitive frailty. A difference (*p*-value for interaction < 0.1) in (bio)marker trajectory between those with and without incident cognitive frailty are indicated by an asterisk. HDL cholesterol, high-density lipoprotein cholesterol; ALT, alanine aminotransferase; GGT, gamma glutamyltransferase; CRP, C-reactive protein. Geometric means are shown for triglycerides, ALT, GGT, and CRP.

In our main model (i.e., model 1), we observed no differences in (bio)marker trajectories between incident cognitively frail men and controls as the trajectories ran parallel and the difference in levels was not statistically significant. In women, we observed differences in the shape of the trajectories of total cholesterol (*p* = 0.067), GGT (*p* = 0.008), and urea (*p* = 0.002) between incident cognitively frail women and controls (Figure 1 and Appendix B Table 1 in Supplementary Material). Total cholesterol increased before women became cognitively frail and decreased after incident cognitive frailty (T_0_), while in controls, total cholesterol levels steadily increased over time. GGT was more or less stable in incident cognitively frail women, while in controls, GGT levels slowly increased from T_−10_ onwards. Urea increased over time in incident cognitively frail women, while in controls, urea levels remained more or less stable.

After further adjustment for level of education and depressive symptoms (model 2), we found no differences in (bio)marker trajectories for men. In women, we found differences in the same biomarker trajectories as in model 1 (i.e., total cholesterol, GGT, urea) and additionally observed a difference in trajectories for ALT in women (*p* = 0.046) (Appendix B Table 1 in Supplementary Material). When further adjusting for BMI and life-style factors (model 3), we still observed no differences in (bio)marker trajectories for men. In women, differences in the trajectories for GGT, urea, and ALT between incident cognitively frail women and controls remained, but the difference in total cholesterol trajectory (*p* = 0.109) was just above our threshold (*p*-value for interaction < 0.1).

To explore the potential impact of loss to follow-up due to mortality on our results, we performed a sensitivity analysis using model 1 in which we excluded participants who died (*n* = 130) during the follow-up period (1993–2012). In men, 61 cognitively frail and 40 controls died. In women, 16 cognitively frail and 13 controls died. Excluding these participants, along with their matched case and/or control(s), resulted in the exclusion of 333 participants in total. After exclusion, in men, the trajectories of total cholesterol (*p* = 0.061) and BMI (*p* = 0.082) differed between cognitively frail men and controls. In women, consistent with the observed differences in the total population, the trajectories of total cholesterol (*p* = 0.066), GGT (*p* = 0.018), and urea (*p* = 0.001) differed between cognitively frail women and controls. In addition, the trajectories of ALT (*p* = 0.057), and albumin (*p* = 0.057) also became different between cognitively frail women and controls (Appendix B Figure 1 in Supplementary Material).

## Discussion

Our aim was to examine whether (bio)marker trajectories differ for those who become cognitively frail compared to those who do not over a follow-up of 15 years. In addition, we hypothesized more unfavorable trajectories for those who became cognitively frail compared to controls. In our main model (model 1), we observed no differences in (bio)marker trajectories between incident cognitively frail men and controls as the trajectories ran parallel and the difference in levels was not statistically significant. In women, we observed differences in the shape of the trajectories of total cholesterol, GGT, and urea between incident cognitively frail women and controls.

Against our expectations, most of the 17 (bio)markers included in this study did not show deviating trajectories between those who became cognitively frail and those who did not. This was, for example, surprising for the inflammation marker CRP, since this biomarker has, longitudinally, been linked to physical frailty (24), cognitive decline (25), and risk of dementia (26, 27), and therefore could serve as a biomarker for cognitive frailty. However, also other epidemiological studies report inconsistent findings regarding longitudinal measures of inflammation markers and cognitive decline (28, 29). In addition, Soysal et al. (30) showed that higher CRP and interleukin-6 (IL-6) levels are cross-sectionally associated to physical frailty, but not longitudinally. Although this study focused on physical frailty, it seems consistent with our null findings regarding cognitive frailty.

We did find a difference in the trajectory for total cholesterol in women. Total cholesterol increased before women became cognitively frail and decreased after incident cognitive frailty (T_0_), while in controls, total cholesterol levels steadily increased over time. In a previous cross-sectional study in MARK-AGE, we also found lower cholesterol levels among people who were already cognitively frail compared to people without frailty (10). Solomon et al. (31) found that non-demented people with high total cholesterol levels around age 50 had poorer cognition 20 years later. In addition, their total cholesterol levels decreased after age 50. The pattern Solomon et al. (31) describe seems comparable to the trajectory we found in incident cognitively frail women. The decline we observed in total cholesterol levels after becoming cognitively frail could be caused by various factors, one of which is medication use. The analyses were adjusted for self-reported use of cholesterol-lowering medication, but information about medication type, dose and therapy compliance were not collected and could therefore not be included in these analyses. Hence, it is unclear whether the trajectories differ due to the occurrence of cognitive frailty, or whether other effects, like a treatment effect, is underlying this difference.

We also observed different urea and GGT trajectories in incident cognitively frail women compared to controls. Higher urea levels can be caused by disrupted blood flow through the kidneys for example through heart failure (32). In contrast, we found lower GGT levels in incident cognitively frail women, while heart failure would also cause increased, and not decreased, GGT levels (33). Lower GGT levels could be caused, for example, by the use of clofibrate, a lipid-lowering agent controlling high cholesterol and triglyceride levels. When adjusting for the use of cholesterol-lowering medication for GGT, results remained similar. It is suggested that serum GGT within the normal range is an early marker for oxidative stress (34). Oxidative stress has been suggested to be associated with frailty (35) and increased GGT levels in later life (80 years and older) were associated with cognitive decline (36). However, we unexpectedly observed lower instead of higher GGT levels in cognitively frail women compared to controls, indicating that cognitive frail women might have less oxidative stress. Overall, there does not seem to be a reasonable explanation for the course of these trajectories. We cannot exclude the possibility that the trajectories of GGT and urea are chance findings.

Differences in (bio)marker trajectories were observed among women, but not among men. We found that more men had poor cognitive functioning compared to women (i.e., more men were identified as incident cognitively frail than women). On the other hand, differences in cognitive functioning between the incident cognitively frail men and their controls were smaller compared to the differences observed in women. Possibly, the women who we identified as incident cognitively frail were relatively worse off than the incident cognitively frail men, having relatively poorer cognitive function and potentially poorer overall health and therefore we only found differences between trajectories for women.

We studied the potential impact of loss to follow-up due to mortality on our results. This was appropriate since some of the trajectories for women indicated a (rapidly) deteriorating health. For example, decreasing cholesterol levels could be caused by malnutrition and increasing urea levels could be caused by heart failure. As expected, mortality rate was higher among those who became cognitively frail compared to those who did not. However, excluding these participants did not materially change the results. In fact, the differences in trajectories for total cholesterol, GGT, and urea in women remained the same and seemed therefore rather robust.

We considered people as being cognitively frail when their global cognitive functioning was poor, given their level of education. Since the prevalence of frailty naturally increases with age, this was not included in the definition of cognitive frailty. The most important difference between our operationalization of cognitive frailty and the classic operationalization for mild cognitive impairment (MCI) (37) is that our definition of cognitive frailty did not include subjective memory complaints. Also, we did not include self-reported activities of daily living which is part of the MCI definition. Recently, a definition for cognitive frailty was proposed combining physical frailty with MCI (38). Since we have previously observed that it is possible to be cognitively frail without being physically frail (10), we defined cognitive frailty only based on cognitive functioning. In our manuscript, the term “cognitive frailty” represents cognitive dysfunction, independent of other (physical) limitations and is only based on poor cognitive functioning given the level of education. We explicitly adjusted for level of education, since highly educated people can also experience cognitive dysfunction and this would otherwise be masked by their cognitive reserves.

One of the strengths of this study is that both cognitive functioning, using a comprehensive neuropsychological test battery, and multiple (bio)markers were objectively measured in four rounds over a follow-up of 15 years, making this a unique cohort for studying the relation between (bio)markers and cognitive functioning over time. In addition, all biomarkers of rounds 2–5 were measured in a single run, limiting inter-assay variation.

This study has some limitations. We tried to include all relevant confounders in the analyses (model 3) but residual confounding may still be present. However, adjustment for confounders had a marginal effect in the results. Further, cognitive functioning was not assessed in participants younger than 45 years. We defined these participants as not being cognitively frail under the assumption that people become cognitively frail with advancing age, mostly from 60 years onwards. In addition, since this population was still relatively young, it could have been too young to find (bio)markers for cognitive frailty. Moreover, since cognitive frailty can be described as a complex syndrome, multiple factors can influence the development of this syndrome. This makes it challenging to identify biomarkers for cognitive frailty. Further, we were unable to exclude participants with dementia. However, given the age-distribution of the cohort, the prevalence and incidence of dementia would be quite low and is therefore unlikely to have influenced our results. Finally, due to the age- and sex-matched study design, the power was limited to analyze differences at any time point. However, more power would probably not lead to other differences in trajectories, because most trajectories run almost parallel and that aspect is unlikely to change with more power.

In conclusion, out of the 17 (bio)markers included in this explorative study, different trajectories between incident cognitively frail women and their controls were found for three biomarkers. However, the relation between these biomarkers and the development of cognitive frailty is unclear. Future studies are needed to confirm these findings. Given the results of this study, we do not yet consider any of the studied (bio)markers as promising (bio)markers for cognitive frailty.

## Ethics Statement

The study was approved by the Medical Ethics Committee of the University Medical Center Utrecht.

## Author Contributions

All authors were involved in interpreting the data, drafting, and approving the manuscript.

### Conflict of Interest Statement

The authors declare that the research was conducted in the absence of any commercial or financial relationships that could be construed as a potential conflict of interest.
