# Supplementary material for: Trajectories of (Bio)markers During the Development of Cognitive Frailty in the Doetinchem Cohort Study
Source: Front Neurol. 2019 May 31;10:497. doi: 10.3389/fneur.2019.00497 (PMC6555275; doi:10.3389/fneur.2019.00497)
Supplement: Supplementary file 1 [file Data_Sheet_1.pdf]

Supplementary material: Appendix A

Biomarkers

Total cholesterol and high-density lipoprotein (HDL) cholesterol were measured until 1998 in non-fasting EDTA-plasma and from 1998 onwards in serum at the Lipid Reference Laboratory of the University Hospital Dijkzigt in Rotterdam, using standardized enzymatic methods. Random plasma glucose was measured with the hexokinase method. In 2013-2014, standardized enzymatic methods (Roche/Hitachi Modular P analyzer, Mannheim, Germany) were used to retrospectively determine biochemical markers from waves 2-5 in non-fasting plasma samples that had been stored at -20 degree Celsius until June 1995 and at -80 degree Celsius from July 1995 onwards. Triglycerides (GPO-PAP assay), alanine aminotransferase (ALT) (kinetic UV assay), gamma-glutamyltransferase (GGT), albumin, and uric acid were measured with a colorimetric method. ALT measurements until June 1995 were recoded as missing (n=2495) because during those years blood plasma was stored at -20 degree Celsius, a temperature at which ALT has poor stability (1). ALT and GGT values greater than three times the upper normal reference were not taken into consideration for the estimation of the trajectories for this particular round since this may indicate liver problems (2). High sensitivity CRP was measured with the principle of particle-enhanced immunological agglutination (Tina-quant CRP). CRP values above 10 mg/L were excluded for the estimation of the trajectories for this particular round because it may indicate an acute-phase response to infection (3). Cystatin C measurement was based on a particle enhanced-turbidimetric immunoassay using reagents from Gentian (Gentian, Moss, Norway) and creatinine was measured with a Creatinine Plus assay (IDMS traceable).

References

1. Williams KM, Williams AE, Kline LM, Dodd RY. Stability of serum alanine aminotransferase activity. Transfusion. 1987;27(5):431-3.
2. Thapa BR, Walia A. Liver function tests and their interpretation. Indian journal of pediatrics. 2007;74(7):663-71.
3. Yeh ET, Willerson JT. Coming of age of C-reactive protein: using inflammation markers in cardiology. Circulation. 2003;107(3):370-1.
