# Supplementary material for: Trajectories of (Bio)markers During the Development of Cognitive Frailty in the Doetinchem Cohort Study
Source: Front Neurol. 2019 May 31;10:497. doi: 10.3389/fneur.2019.00497 (PMC6555275; doi:10.3389/fneur.2019.00497)
Supplement: Supplementary file 2 [file Data_Sheet_2.pdf]

Supplementary material: Appendix B

Table 1. Differences in (bio)marker trajectories between those with and without incident cognitive frailty, stratified by sex.

**Markers and biomarkers**	**Men**						**Women**					
	**Model 1**		**Model 2**		**Model 3**		**Model 1**		**Model 2**		**Model 3**	
	**$\chi 2$**	**Pr > ChiSq**	**$\chi 2$**	**Pr > ChiSq**	**$\chi 2$**	**Pr > ChiSq**	**$\chi 2$**	**Pr > ChiSq**	**$\chi 2$**	**Pr > ChiSq**	**$\chi 2$**	**Pr > ChiSq**
Self-reported health	9.22	0.101	1.93	0.858	1.39	0.926	3.55	0.616	2.91	0.714	2.78	0.734
BMI	2.28	0.809	3.21	0.668	4.36	0.499	2.22	0.818	0.71	0.983	0.51	0.992
Waist circumference	2.79	0.733	2.79	0.733	3.17	0.673	2.12	0.833	2.42	0.788	3.58	0.612
Systolic blood pressure	1.43	0.921	0.95	0.966	0.93	0.968	4.04	0.544	3.11	0.683	2.45	0.785
Diastolic blood pressure	1.97	0.854	2.93	0.712	3.60	0.609	1.38	0.927	0.56	0.990	0.63	0.987
Total cholesterol	8.17	0.147	7.22	0.205	7.37	0.195	**10.32**	**0.067**	**10.59**	**0.060**	9.01	0.109
HDL cholesterol	8.83	0.116	5.92	0.314	5.15	0.398	1.64	0.897	1.98	0.853	2.45	0.784
Glucose	2.66	0.752	3.46	0.629	3.63	0.604	4.02	0.547	3.93	0.559	4.27	0.511
Triglycerides	5.07	0.408	5.36	0.373	4.66	0.459	3.56	0.614	2.36	0.797	2.32	0.803
ALT	6.64	0.249	5.77	0.330	5.87	0.319	8.17	0.147	**11.29**	**0.046**	**12.96**	**0.024**
GGT	2.31	0.804	2.23	0.817	2.34	0.800	**15.71**	**0.008**	**17.48**	**0.004**	**20.05**	**0.001**
CRP	5.02	0.413	5.91	0.315	4.62	0.465	6.96	0.223	7.31	0.199	8.36	0.138

Albumin	7.34	0.196	6.27	0.281	6.54	0.257	8.18	0.147	6.70	0.244	6.10	0.297
Cystatin C	3.35	0.646	2.35	0.799	1.96	0.855	6.63	0.250	5.91	0.315	4.47	0.484
Creatinine	0.96	0.966	2.47	0.781	2.41	0.790	4.24	0.516	5.00	0.416	5.42	0.367
Uric Acid	2.45	0.784	1.90	0.863	1.88	0.866	4.86	0.433	6.42	0.267	5.74	0.333
Urea	1.18	0.946	2.69	0.747	2.87	0.720	**19.08**	**0.002**	**17.14**	**0.004**	**14.60**	**0.012**

Model 1: age (linear and quadratic), examination round, and time

Model 2: age (linear and quadratic), examination round, time, level of education, and depressive symptoms

Model 3: age (linear and quadratic), examination round, time, level of education, depressive symptoms, BMI, smoking, alcohol intake, and physical activity. Note: The trajectories of BMI and waist circumference were not adjusted for BMI.

Additional adjustment in all three models: Trajectories of systolic and diastolic blood pressure were adjusted for anti-hypertensive medication, trajectories of total cholesterol, HDL cholesterol and triglycerides were adjusted for cholesterol-lowering medication, and trajectories of glucose were adjusted for the use of glucose-lowering medication;

A difference (p-value for interaction<0.1) in (bio)marker trajectory between those with and without cognitive frailty is indicated in bold;

Abbreviations: BMI, body mass index; HDL cholesterol, high-density lipoprotein cholesterol; ALT, alanine aminotransferase; GGT, gamma glutamyltransferase; CRP, C-reactive protein.

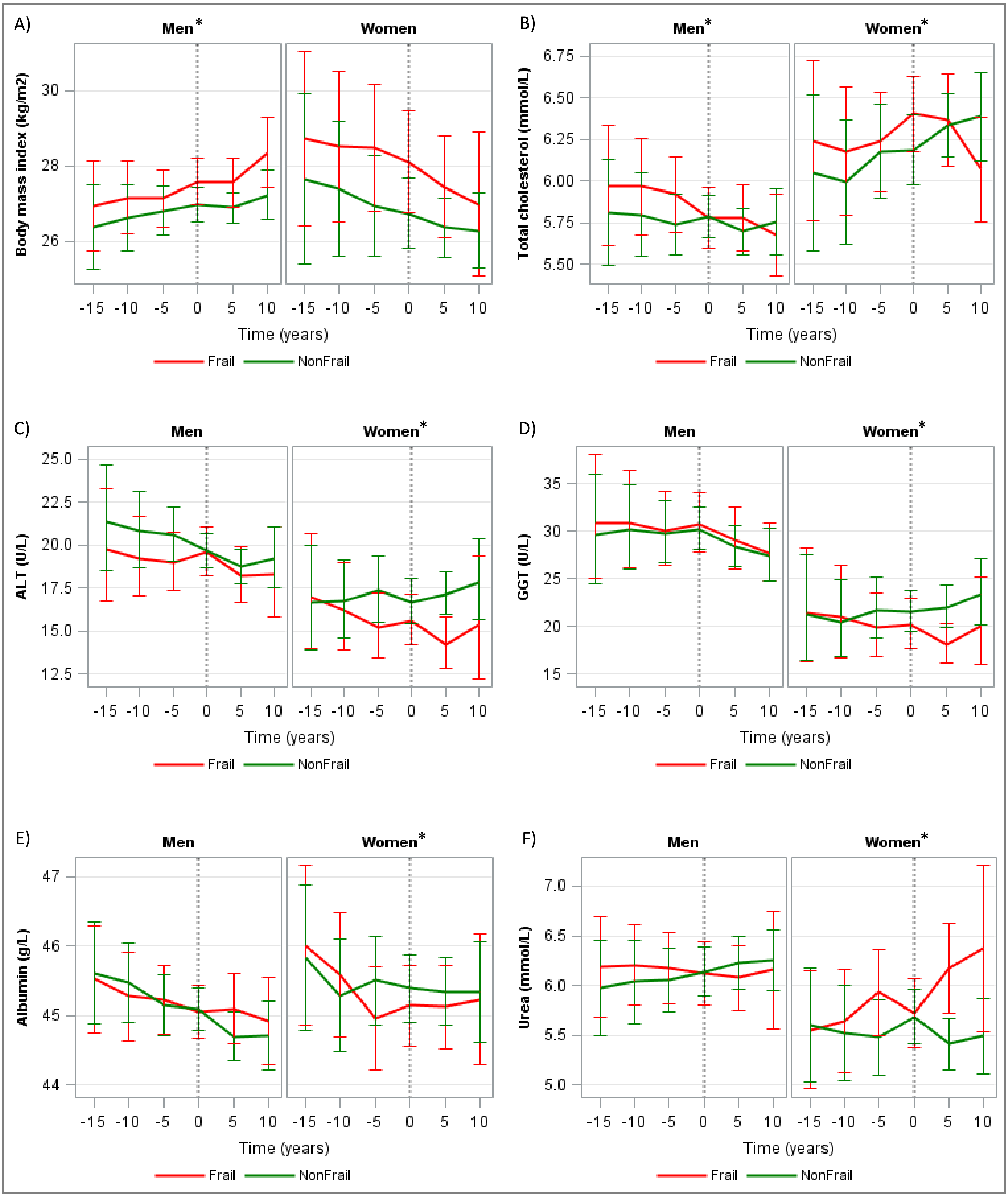

A)
Men*
Women
Body mass index (kg/m2)
B)
Men*
Women*
Total cholesterol (mmol/L)
C)
Men
Women*
ALT (U/L)
D)
Men
Women*
GGT (U/L)
E)
Men
Women*
Albumin (g/L)
F)
Men
Women*
Urea (mmol/L)
Time (years)
Frail
NonFrail

Figure 1. Sensitivity analysis for loss to follow-up due to mortality.

Participants who died during the follow-up period (1993-2012) along with their matched case and/or control(s) were excluded in these analyses (n=333). Trajectories are shown of body mass index (A), total cholesterol (B), ALT (C), GGT (D), albumin (E), and urea (F) of incident cognitively frail people (red lines) and controls (green lines) with 95% confidence intervals stratified by sex and corrected for age and, if appropriate, medication use (model 1), where men and women were hypothetically 60 years old at the time of incident cognitive frailty. A difference (p-value for interaction<0.1) in (bio)marker trajectory between those with and without incident cognitive frailty are indicated by an asterisk.

Abbreviations: ALT, alanine aminotransferase; GGT, gamma glutamyltransferase. Geometric means are shown for ALT and GGT.
